# Prevalence of HIV-associated ophthalmic disease among patients enrolling for antiretroviral treatment in India: A cross-sectional study

**DOI:** 10.1186/1471-2334-9-158

**Published:** 2009-09-23

**Authors:** Sophia Pathai, Alaka Deshpande, Clare Gilbert, Stephen D Lawn

**Affiliations:** 1 International Centre for Eye Health, London School of Hygiene & Tropical Medicine, London, UK; 2 ART Center, Department of HIV Medicine, Sir JJ Hospital, Byculla, Mumbai, India; 3 Desmond Tutu HIV Centre, Institute for Infectious Disease and Molecular Medicine, Faculty of Health Sciences, University of Cape Town, Cape Town, South Africa; 4 Clinical Research Unit, Department of infectious and Tropical Diseases, London School of Hygiene & Tropical Medicine, London, UK

## Abstract

**Background:**

The ocular manifestations of HIV may lead to visual impairment or blindness. In India, patients typically initiate antiretroviral treatment (ART) with low CD4 cell counts when the risk of ocular complications may be high. The objective of this study was to determine the prevalence and types of HIV-associated ocular conditions in patients referred for ART in India.

**Methods:**

This cross-sectional study was undertaken at a large public sector ART centre in Mumbai, India. Data collection including a standardised symptom screen, and an ophthalmic examination were performed on all consecutive patients satisfying the criteria for enrolment into the ART clinic irrespective of the presence or absence of ophthalmic/visual symptoms.

**Results:**

Enrolled patients (n = 149) had a median CD4 cell count of 180 cell/μL (inter-quartile range [IQR], 106-253 cells/μL). The prevalence of HIV-associated ocular disease was 17.5% (95% CI, 11.2-23.6%) in all participants and 23.8% (95% CI: 14.5-33.1) in those with CD4 cell counts <200 cells/μL (n = 84). Only 7.7% of patients with HIV-associated ocular disease reported any eye symptoms in the standardised symptom screen. Objective visual impairment was detected in 20% of those with HIV-associated ocular disease compared to 6% in those without ocular manifestations (p = 0.02). Vitreoretinal disease was the most common manifestation, of which cytomegalovirus retinitis (CMVR) was the most frequent retinal infection (overall prevalence 8.7%, 95% CI: 4.1-13.3%). In a multivariable analysis, HIV-associated ocular disease was independently associated with a CD4 count <100 cells/μL (odds ratio [OR], 6.3, 95% CI: 1.5-25.9) and WHO clinical stages 3 and 4 (OR 9.4, 95% CI: 2.4-37.2). However, symptoms were not independently predictive of ocular disease. Sensitivity of ocular symptom screening was 7.7%, with a positive predictive value of 18% in this population.

**Conclusion:**

Over a fifth of unselected patients who are eligible for ART in this setting have HIV-related ocular disease of which CMVR is the most common form. Such patients may be at risk of developing ocular immune reconstitution phenomena during ART. Screening for ocular symptoms is not a reliable method to identify those with ocular morbidity and this highlights the need for routine ophthalmic screening prior to commencement of ART.

## Background

HIV-related eye disease may affect 50-75% of HIV-infected people worldwide at some point during the course of their illness [1]. This generally takes the form of opportunistic infections that can affect any of the ocular tissues, from the eyelids to the retina. In particular, those conditions affecting the retina may lead to chronic visual impairment or blindness. The spectrum of HIV-related disease appears to differ by geographic location, with reports suggesting that infection-related retinitis is not as common in sub-Saharan Africa compared to industrialised countries and South Asia [2-4]. The very low prevalence of CMVR seen in sub-Saharan Africa may not be a direct reflection of lower incidence, but possibly reflect that these patients die from systemic opportunistic infections before their CD4 counts fall low enough to allow the development of HIV-related eye disease [2,3,5]. In sub-Saharan Africa, anterior segment conditions such as herpes zoster ophthalmicus are more common than posterior segment (retinal) opportunistic infections [3]. The situation is less clear in South Asia - ocular manifestations of HIV in India were first reported in 1995 [4]. Since then, the number of patients affected by HIV has greatly increased, and the patient demographic is likely to have changed.

The immune reconstitution inflammatory syndrome (IRIS) is a well recognised complication of antiretroviral therapy (ART), which may lead to the clinical deterioration of opportunistic infections due to the rapid restoration of immunological host responses during the initial weeks of treatment [6]. Immune recovery uveitis (IRU) is the predominant form of ocular IRIS and mainly occurs in patients with pre-existing CMVR at the time of ART initiation [7]. IRU is characterised by ocular inflammation following ART initiation and can result in visual loss from macular oedema, retinal neovascularisation and cataract [7,8]. With increasing availability of antiretroviral therapy, IRU may play a significant role in contributing to ocular morbidity. Identification and treatment of ocular disease prior to ART initiation is, therefore, important.

Current estimates suggest that between 2-3.1 million people are diagnosed with HIV in India [9]. The Government of India launched a free public sector ART initiative in 2004 and access to ART is steadily increasing [10]. The estimated prevalence of HIV-related eye disease in India is reported to be between 8-45% [4,11,12], but population demographics were different in each study making comparisons difficult. Data are scarce concerning the burden of ocular disease in patients initiating ART from low CD4 counts in resource-limited countries, who are likely to be at high risk of ocular opportunistic infections. Previous prevalence estimates have generally been within populations selected on the basis of symptoms or ophthalmological referral. To our knowledge, there have been no studies to date in the Indian sub-continent that have evaluated the prevalence of HIV-related ocular lesions in an unselected, ART-naïve population. The aim of this study was to conduct an epidemiological study of HIV-related eye-disease (in particular vitreoretinal disease) in all HIV-infected adults, prior to commencing ART attending a large HIV referral and ART centre in Mumbai, India.

## Methods

### Study setting and patients

This cross-sectional study was carried out at the ART Centre, Sir JJ Hospital, Mumbai - a tertiary referral centre and teaching hospital. The ART centre is recognised as a National AIDS Control Organisation (NACO) ART Centre of Excellence. It is the largest ART centre in India and provides free ART medication to all registered clinic attendees. ART is initiated in patients with CD4 counts <250 cells/μL or with a prior AIDS diagnosis (WHO clinical stage 4 disease), according to NACO guidelines [13]. All patients attending the clinic over a 2-month period were recruited irrespective of ophthalmic symptoms or history or CD4 count. Demographic and medical information was obtained by a combination of direct questioning by the ART clinic counsellor, and review of the medical case notes. Data obtained included mode of transmission, present CD4 count, WHO clinical stage and duration since HIV diagnosis. None of this information was disclosed to personnel conducting the ophthalmic examination. In addition, standardised symptom screening was conducted as related to ocular pathology; this included questions relating to impairment of vision, ocular pain and presence of floaters.

### Ophthalmic examinations

Ophthalmic examinations were conducted by an ophthalmologist. Presenting and best-corrected visual acuity was measured using Log MAR (logarithm of the minimal angle of resolution) visual acuity charts, which have been employed in previous blindness prevalence studies [14]. Visual impairment and blindness was defined as per recently revised WHO guidelines, where visual impairment is defined as presenting visual acuity of less then 6/18 (0.3 Log MAR), but equal to or better than 3/60 (0.05), and blindness as presenting visual acuity of 3/60 or worse, in the better eye [15]. Ophthalmic diagnoses were made clinically based upon slit lamp biomicroscopic examination of the anterior segment (eyelids, conjunctiva, cornea, anterior chamber), and dilated indirect ophthalmoscopy to provide views of the central and peripheral retina. One in four examinations were randomly repeated by another eye care professional; diagnoses were compared after both examinations were completed and any disagreements were discussed to arrive at a final diagnosis. In addition any ophthalmic diagnoses available from the medical cased notes were documented but not revealed to the examiner(s) prior to examination. The diagnosis of HIV-related eye conditions such as cytomegalovirus retinitis (CMVR) was based upon characteristic clinical features using standardised descriptions [16]. All participants with active ocular disease (whether or not related to HIV) were referred to the Ophthalmology Department within Sir JJ Hospital for further assessment and/or treatment.

### Ethics Statement

This study was conducted according to the principles expressed in the Declaration of Helsinki. The study was approved by the Institutional Review Board of Sir JJ Hospital, and the London School of Hygiene & Tropical Medicine Ethics Committee (Ref 5388). Adults who were about to commence ART were recruited for this study after obtaining written informed consent.

### Data analysis

Information was recorded on a pre-coded data collection form. All statistical analyses were performed using Stata 10.0IC (Stata Corp. College Station, TX, USA). In analyses, chi-squared tests (and Fisher's exact test where appropriate) were used to compare proportions, and the Wilcoxon rank-sum test to compare medians. Multiple logistic regression was performed to examine associations between ocular HIV manifestations and patient demographic and clinical characteristics. All P-values reported were two-sided at a 5% significance level.

## Results

### Study population

One hundred and forty-nine consecutive HIV-infected participants (298 eyes) were examined (Table 1). All participants were of Indian origin, and 93.2% were resident in Mumbai. The median age of the 149 patients was 36 years (range: 16-65 years), and 104 patients were male (69.8%). HIV infection was acquired through heterosexual contact, homosexual contact, blood transfusion and intravenous drug use in 139 (94.6%), 4 (2.7%), 2 (1.3%) and 2 participants (1.3%), respectively. The main sources of referral to the ART Centre were private clinics where patients had undergone HIV testing upon relevant history/examination findings - patients were thus referred to the ART Centre to receive free ART and HIV management. Patients were also referred internally from other specialty departments within Sir JJ Hospital. The median CD4 cell count of participants was 180 cells/μL (interquartile range [IQR], 106-253 cells/μL; range, 18 - 554 cells/μL). 59.7% (89) participants had symptomatic disease classified as WHO clinical stages 3 and 4. Over half (53.0%) of the sample had either current or a previous history of tuberculosis (pulmonary and extra-pulmonary).

**Table 1:** Characteristics of study participants who did or did not have HIV-related ocular disease (n = 149, unless otherwise specified)

Characteristic	Participants with HIV-related ocular conditions (%) (n = 26)	Participants without HIV related eye conditions (%) (n = 123)	P-value
**Median age (n = 148)**			
Years	38	36	0.95
			
**Gender**			
Male [n(%)]	18 (69.2)	86 (69.9)	0.94
			
**Marital status**			
Single	3 (11.5)	13 (10.6)	0.81
Married	20 (76.9)	88 (70.7)	
Divorced	0	2 (1.6)	
Widowed	3 (11.6)	21 (17.1)	
			
**Occupation**			
None	13 (50.0)	55 (44.7)	0.44
Unskilled	2 (7.7)	22 (17.9)	
Skilled	11(42.3)	46 (37.4)	
			
**Exposure (n = 147)**			
Heterosexual	26 (100.0)	113 (93.4)	0.99
Homosexual	0	4 (3.3)	
Blood transfusion	0	2 (1.7)	
Intravenous drug use	0	2 (1.7)	
			
**Median CD4 count**			
Cells/μL	106	200	0.003
			
**CD4 count (cells/μL)**			
0-50	7 (26.9)	6 (4.9)	0.003
51-100	6 (23.1)	17 (13.8)	
101-150	5 (19.2)	18 (14.6)	
151-200	2 (7.7)	23 (18.7)	
201-250	2 (7.7)	25 (20.3)	
>250	4 (15.4)	34 (27.6)	
			
**WHO clinical stage**			
1/2	2 (7.8)	58(47.2)	<0.001
3/4	24 (92.3)	65 (52.8)	
			
**Months since HIV diagnosis**			
≤ 12	11 (42.3)	51 (42.5)	0.94
13-24	6 (23.1)	27 (22.5)	
25-60	5 (19.2)	28 (23.3)	
>61	4 (15.4)	14 (11.7)	
			
**Tuberculosis status**			
No history	7 (26.9)	63 (51.2)	0.07
Past history	11 (42.3)	31 (25.2)	
Current	8 (30.8)	29 (23.6)	
			
**History of eye symptoms (n = 148)**			
None	24 (92.3)	113 (92.6)	0.82
Reduced vision	2 (7.7)	6 (4.9)	
Pain	0	2 (1.6)	
Floaters	0	1 (0.8)	
			
**Visual acuity (n = 142)**			
None	20 (80.0)	110 (94.0)	0.02
Visual impairment	4 (16.0)	7 (5.6)	
Blind	1 (4.0)	0	

### Ocular manifestations

Overall 7.4% (95% confidence interval [95% CI] 3.8-12.9%) (n = 11) of the participants complained of any eye-related symptoms upon direct questioning; this was mainly related to blurred vision/reduced visual acuity. Overall, the median presenting visual acuity was 6/9 in both eyes, ranging from 6/5 to no perception of light (NPL). The prevalence of any visual impairment (including blindness) was 8.5%. (95% CI: 3.8-13.1%) (n = 12). This included patients with known pre-existing eye disease - 5 participants had uncorrected refractive error, one participant had cataracts (age-related), and one had visual impairment from long-standing ocular trauma.

Ophthalmic manifestations of HIV were detected in 26 patients, giving a prevalence of 17.5% (95% CI: 11.2-23.6%). Of these only 2 (7.7%) patients had ophthalmic symptoms (blurred vision, eye pain, floaters). In participants with no ocular manifestations of HIV, the prevalence of any visual impairment was 6.0% compared to 20% in those who did have ocular manifestations (p = 0.02). 4 patients had visual impairment and 1 was blind in those with HIV-associated ocular disease.

Table 2 shows the overall prevalence of ocular disease and cause-specific posterior segment (vitreoretinal) disease within the total study population and within the sub-set of patients with CD4 counts <200/μL. The most common ocular manifestation was CMV retinitis (CMVR), which was present in 13 cases (overall prevalence 8.7%, 95% CI: 4.1-13.3%), Involvement was bilateral in 7/13 cases; there was one retinal detachment related to CMVR. Four of the participants with CMVR had visual impairment or blindness but only one patient reported this as a symptom.

**Table 2:** Prevalence of ocular disease and posterior segment infections, by CD4 counts.

Condition	No of cases: CD4 count <200 cells/μL (n = 84)	Prevalence (95% CI)	No of cases: All CD4 counts (n = 149)	Prevalence (95% CI)
Any ocular condition	18	23.8% (14.5-33.1)	26	17.5% (11.2-23.6)
Vitreoretinal infection (CMVR & TB)	13	15.5% (7.6-23.3)	17	11.4% (6.2-16.6)
CMVR	10	11.9% (4.8-19.0)	13	8.7% (4.1-13.3)
TB choroiditis	3	3.7% (0.04-7.6)	4	2.7% (0.05-5.3)
HIV retinopathy	7	8.7% (2.2-14.3)	7	4.7% (1.3-8.1)

HIV retinopathy, as manifested by cotton-wool spots and retinal haemorrhages, was the second most common ophthalmic manifestation, affecting 7 participants, giving a prevalence of 4.7% (95% CI: 1.3-8.1%) [Table 2]. Disease was bilateral in all cases and there was impairment of visual acuity in one case with macular oedema. Choroiditis was diagnosed in 4 participants; lesions were focal and spared the macula. Tubercular choroiditis was diagnosed in 4 cases and all occurred in patients with either a past history (n = 2) or current history (n = 2) of systemic tuberculosis. Neuro-ophthalmic complications included optic atrophy (1 case, unilateral), and optic disc swelling (1 case, bilateral). Anterior segment complications occurred in two patients - one had bilateral eyelid molluscum contagiosum, and one had unilateral herpes zoster affecting the eyelid and forehead (but without corneal involvement). There were no ocular lesions suggestive of Kaposi's sarcoma.

### Risk Factors for HIV-Associated Eye Disease

Table 1 shows the characteristics of groups of patients with and without HIV-associated eye disease. There were no significant differences between the two groups in gender, age, marital status, occupation and duration since HIV diagnosis. However, patients with eye disease had more advanced immunodeficiency as reflected by significantly lower CD4 cell counts and more advanced WHO stage of disease. Overall the prevalence of eye disease among patients with CD4 cell counts ≤ 200 cells/μL was 23.8% (95% CI: 14.5-33.1%), compared to 9.2% (95% CI: 2.0-16.4%), among those with CD4 cell counts ≤ 200 cells/μL (p = 0.02). There was also a strong trend for a current or previous history of TB to be associated with eye disease (Table 1). Although patients with eye disease were significantly more likely to have impaired visual acuity, there was no difference in the frequency of reported symptoms between the two groups (Table 1).

Analysis of the distribution of the various ocular manifestations of HIV by CD4 count revealed that all participants who were diagnosed with CMVR, and who were ART-naïve at the time of the study had CD4 counts ≤ 200 cells/μL. (3/13 CMVR cases were previously treated with ART by private physicians, and had CD4 counts >200 cells/μL at their time of entry to our study). The median CD4 count was 138 cells/μL, (IQR = 67.5-188.5 cells/μL; range = 30-554 cells/μL) and all patients with *active *CMVR had CD4 counts <200/μL. The majority (71.4%) of participants with HIV retinopathy were categorised at WHO clinical stage 3 or 4, and all had CD4 counts ≤ 150 cells/μL. The median CD4 count in cases of tubercular choroiditis was 29 cells/μL. Overall, 76.9% of the study population with HIV-associated ocular conditions had CD4 counts ≤ 200 cells/μL.

Multivariable analysis, adjusting for baseline characteristics significant at a 5% significance level and *a priori *confounders, showed that a low CD4 count (≤ 100 cells/μL), and WHO clinical stages 3 and 4 were independently associated with increased odds of having an ocular manifestation of HIV (Table 3). (The presence of systemic TB was initially included as a co-variate in the regression model, but due to its high correlation with WHO clinical stage, both parameters could not be fitted and thus only WHO clinical stage was retained due to its strong clinical and statistical association with ocular disease).

**Table 3:** Multivariable analysis for risk factors for ocular manifestations of HIV, among patients prior to commencing ART (n = 135).

Variable	Odds ratio (OR) for ocular manifestation of HIV	95% CI	P-value
**Age group (years)**			
16-30	1	-	0.2
31-35	0.1	0.02-0.7	
36-40	0.6	0.1-2.5	
41-45	0.7	0.1-3.3	
>46	0.6	0.1-3.2	
			
**Sex**			
Male	1	-	0.67
Female	1.3	0.4-5.0	
			
**CD4 count (cells/μL)**			
>201	1	-	0.02
101-200	1.3	0.3-5.4	
0-100	6.3	1.5-26.3	
			
**WHO clinical stage**			
1 or 2	1	-	0.001
3 or 4	9.4	2.4-37.2	
			
**Visual acuity**			
Normal	1	-	0.14
Visual impairment/blind	3.1	0.7-14.0	
			
**Ocular symptoms**			
No	1	-	0.95
Yes	1.1	0.1-7.8	
			
**Duration of disease (months)**			
≤ 12			
13-24	1	-	0.8
25-60	0.9	0.2-3.4	
>60	0.5	0.1-2.2	
	0.5	0.1-3.0	

### Sensitivity and specificity analyses

Ocular symptoms (ascertained using the standardised symptom screening questionnaire) were not independently predictive of ocular disease and although the specificity for HIV-related eye disease was high (92%; 95%CI: 86.1-96.3%), the sensitivity and positive predictive value were very low (7.7%, 95% CI: 1.3-26.6% and 18%, 95%CI: 3.2-52.2%, respectively) (Table 4). Analyses were conducted to ascertain the proportion of participants with HIV ocular complications that would be detected if only subsets of higher risk (e.g. low C4 count, history of TB) were screened. Using WHO clinical stage 3/4 as a screening subset for HIV eye disease led to a sensitivity of 92.3% (95% CI: 73.4-98.6%), and specificity of 52.8% (95% CI: 43.7-61.8%). There was no clear CD4 count threshold above which led to a marked increment in sensitivity. With decreasing CD4 counts, specificity and PPV increased (Table 4). The number needed to screen (NNS) is the number of people that need to be screened to detect one new case of eye disease. The lowest NNS was found using vision status, screening those with visual impairment in one eye gave a NNS of 2.4 (95%CI: 1.4-6.0). Overall, the NNS was lower the more advanced the degree of immunodeficiency as manifested by CD4 count and WHO clinical status.

**Table 4:** Sensitivity/specificity analysis using CD4 count thresholds, WHO clinical status, TB status, presence of ocular symptoms, presence of visual impairment

Factor	Number with HIV eye disease (n = 26)	Number without HIV eye disease	Sensitivity	Specificity	Positive predictive value	Number needed to screen* (95%CI)
		(n = 123)	(95% CI)	(95% CI)	(95% CI)	
WHO Clinical status						
Stage 1/2	2	58	7.7 (1.3-26.6)	52.8 (43.7-61.8)	3.3 (0.6-12.5)	30.3 (8-166.7)
Stage 3/4	24	65	92.3 (73.4-98.6)	47.2 (38.2-56.3)	27.0 (18.3-37.6)	3.7 (2.9-5.5)
TB status						
Past/current TB	19	60	73.1 (52.0-87.6)	51.2 (42.0-60.2)	24.1 (15.4-35.2)	4.1 (2.9-6.5)
CD4 count (cells/μL)						
≤ 50	7	6	27.0 (12.4-48.0)	95.1 (89.2-98.0)	53.8 (26.1-78.0)	1.9 (1.3-3.8)
≤ 100	13	23	50.0 (30.4-69.6)	81.3 (73.1-87.5)	36.1 (21.3-53.8)	2.8 (1.9-4.7)
≤ 150	18	41	69.2 (48.1-84.9)	66.7 (57.5-74.8)	30.5 (19.5-44.0)	3.3 (2.3-5.1)
≤ 200	20	64	76.9 (55.9-90.2)	48.0 (38.9-57.1)	23.9 (15.5-34.6)	4.2 (2.9-6.5)
≤ 250	22	89	84.6 (64.3-95.0)	27.6 (20.1-36.6)	19.8 (13.1-28.7)	5.1 (3.5-7.6)
Ocular symptoms						
Present	2	9	7.7 (4.0-13.2)	92.6 (86.1-96.3)	18.1 (3.2-52.2)	5.5 (1.9-31.2)
Vision status						
Visual impairment	5	7	20.0 (7.6-41.3)	94.0 (87.6-97.3)	41.7 (16.5-71.4)	2.4 (1.4-6.0)

*The number needed to screen (NNS) is the number of people that need to be screened to detect one new case of eye disease

## Discussion

This study evaluated the prevalence of ocular disease in HIV-infected patients referred to an ART service in India, regardless of whether patients reported ocular or visual symptoms. Ophthalmic manifestations of HIV were found in over a fifth of patients and the most common manifestations were due to opportunistic vitreoretinal infections, particularly CMVR. These findings are important with regard to policies for screening for ocular disease prior to ART. Screening in this patient population is particularly important not only because of the high prevalence of disease in this highly immunocompromised patient group but also in view of the potential for worsening of ocular disease due to ART-induced IRIS.

The overall prevalence of HIV-associated ocular disease in this population was 17.5%, and 23.4% in those with CD4 counts <200 cells/μL. Other studies in India have reported prevalence estimates ranging between 8 and 45%, with study numbers ranging from 100-112 participants. Higher prevalence estimates in other studies may result from the study of patients referred to a tertiary ophthalmic centre, evaluation of patients based upon symptoms or inclusion of patients after initiation of ART in whom complications of IRIS have been present [4,11,12]. Our study is the largest prevalence study of HIV ocular manifestations in India to date, and the participants in our study are likely to be a representative sample of ART-naïve HIV-infected persons presenting for ART treatment and care within a large Indian metropolis. A key strength of this study is that all patients were examined regardless of the presence or absence of ocular symptoms.

Our findings are consistent with other prevalence studies in Indian populations of HIV-infected patients in whom retinal/posterior segment disease is the most common finding [4,11,12]. CMVR continues to be the most common ocular finding in India, and may affect up to 11.9% of patients with CD4 counts <200 cells/μL. With the high prevalence (73.1%) of systemic TB (either past or current) in the study population, it is not surprising that cases of tubercular choroiditis were also seen. All patients with ocular (choroidal) TB had a co-existent history of systemic TB. This may have implications in terms of any subsequent immune reconstitution-mediated phenomena that are strongly associated with disseminated forms of TB [17]. We found that 15.5% of patients with CD4 counts <200 cells/μL had retinal infections which are known to predispose to immune recovery uveitis (IRU). Thus, as access to ART expands nationwide, Indian patients may be at high risk of visual loss upon commencement of ART, whether due to the direct adverse effects of opportunistic disease, or immune-reconstitution mediated-phenomena. Future prospective studies are needed to examine this.

There were very few cases of anterior segment disease (eyelids, conjunctiva, cornea), which is in keeping with findings from other Indian studies [4,11]. There were no ocular lesions of Kaposi's sarcoma in this sample which is in agreement with the very low prevalence of AIDS-related malignancies in South Asia [18], and is thought to be due to low seroprevalence of human herpes virus-8 (HHV-8) in these populations [19].

The prevalence of ophthalmic lesions associated with HIV was significantly higher in patients with CD4 counts between 0-100 cells/μL, and in those with WHO clinical stages 3 or 4 (Table 1). We found that those with CD4 counts <100 cells/μL were six times more likely to have an ocular HIV-related lesion, and those with advanced HIV as defined by WHO clinical stage were nine times more likely to be at risk, after adjustment for potential confounders (Table 3). Thus CD4 count and WHO clinical stage may be important predictors of the presence of HIV-related eye disease in an ART-naïve population. Our finding that 76.9% of patients with ocular manifestations of HIV had CD4 counts <200 cells/μL is comparable with the results of the one other study in which CD4 cell count measurements were available [11].

It is interesting to note that out of 12 patients with visual impairment in the overall study sample, only one complained of reduced visual acuity. In addition, of these 12 patients, 5 had non HIV-related treatable/correctable conditions. The reporting of ocular symptoms was low (<10%) in groups of patients with and without ocular disease. This may reflect genuine non-report due to symptoms not being perceived by patients as important. Alternatively, it has been suggested that patients with HIV may not seek treatment for ocular conditions due a perceived stigma related to HIV [12,20]. Despite similar reporting of ophthalmic symptoms between the two groups, patients with HIV-associated eye disease were significantly more likely to have visual acuities consistent with blindness or visual impairment than the group without ocular conditions. The poor association between ocular disease and ocular symptoms is important to note as often patients will seek ophthalmic examination only if they have visual complaints or symptoms. The combination of visual impairment and HIV infection may lead to increased economic dependency for the individual and their family, as well as a psychosocial impact with loss of self-esteem and possible family neglect [20].

## Conclusion

Current practice at several ART centres in India is to refer patients for an ophthalmic examination only upon complaint of ocular symptoms or after a physician has noted abnormal ocular signs on medical examination. Our findings highlight the need for routine baseline ophthalmic screening at the pre-ART stage. The findings of this study, particularly the low sensitivity and PPV of ocular symptom screening in this population suggest that utilising patients' subjective description of ocular complaints is not a reliable method for detecting HIV-associated ocular disease. However, detection of CMVR using a symptom-oriented approach has produced diverse findings. In the USA, studies suggest the percentage of individuals with CMVR reporting ocular symptoms lies anywhere between 23-88% [21-23], and in South East Asia, the estimate is between 31-44% [24].

Routine baseline ophthalmic screening of all patients may be particularly important for conditions such as CMVR or TB choroiditis, where if a lesion is peripherally located within the retina the patient may not experience reduction in visual acuity, or may not place significance upon the presence of floaters or other ocular symptoms. If such a lesion is not detected and treated at an early stage it may extend to the posterior pole (optic disc and macula), leading to potentially blinding complications either due to the direct pathological effects of opportunistic disease, or due immune-reconstitution mediated-phenomena once ART is commenced.

Further work is indicated to formally evaluate the validity of patients' symptoms in detecting different types of HIV-associated ocular disease. It is possible that specificity and sensitivity using symptoms as a screen for detecting ocular diseases varies by anatomical location (i.e. anterior segment vs. posterior segment), and this information may be of value in resource-limited settings where a full ophthalmic evaluation in every patient is not possible. Conducting a full ophthalmic examination requires trained personnel and adequate resources that may be difficult or impossible to provide for all patients in resource-limited countries. However, where possible, we suggest that an ophthalmic examination prior to commencing ART is of value in order to reduce HIV-related ocular morbidity in the era of increased ART availability in India. HIV treatment programs in resource-poor settings are unlikely to provide ophthalmic screening for all patients. The results of our sensitivity and specificity analyses suggest that ophthalmic screening examinations might be prioritised for subsets of patients with advanced immunodeficiency as manifested by WHO clinical status 3 or 4, and the presence of systemic TB.

With the advent of increased ART availability, life expectancy for HIV-infected persons is likely to improve. However, the burden of HIV-related ocular disease is likely to remain stable or even increase with possible immune reconstitution ocular complications. Strategies for screening high-risk populations for HIV-related ocular disease are needed as well as provision for the management and treatment of these conditions once detected.

## Competing interests

The authors declare that they have no competing interests.

## Authors' contributions

SP conceived the study, and participated in its design and coordination, performed statistical analyses, and drafted the manuscript. SP and AD conducted the fieldwork. CG and SDL participated in the design of the study and helped to draft the manuscript. All authors read and approved the final manuscript.

## Pre-publication history

The pre-publication history for this paper can be accessed here:

http://www.biomedcentral.com/1471-2334/9/158/prepub

## References

[B1] Kestelyn PG Cunningham ET Jr HIV/AIDS and blindness Bull World Health Organ 2001 79 208 213 11285664 PMC2566369

[B2] Jaffar S Ariyoshi K Frith P Okouchi Y Sabally S Ajewole T Bailey R Lee PS Corrah T Johnson G Retinal manifestations of HIV-1 and HIV-2 infections among hospital patients in The Gambia, west Africa Trop Med Int Health 1999 4 487 492 10.1046/j.1365-3156.1999.00425.x 10470340

[B3] Lewallen S Kumwenda J Maher D Harries AD Retinal findings in Malawian patients with AIDS Br J Ophthalmol 1994 78 757 759 10.1136/bjo.78.10.757 7803351 PMC504929

[B4] Biswas J Madhavan HN George AE Kumarasamy N Solomon S Ocular lesions associated with HIV infection in India: a series of 100 consecutive patients evaluated at a referral center Am J Ophthalmol 2000 129 9 15 10.1016/S0002-9394(99)00415-8 10653406

[B5] Kestelyn P The epidemiology of CMV retinitis in Africa Ocul Immunol Inflamm 1999 7 173 177 10.1076/ocii.7.3.173.4002 10611725

[B6] French MA Lenzo N John M Mallal SA McKinnon EJ James IR Price P Flexman JP Tay-Kearney ML Immune restoration disease after the treatment of immunodeficient HIV-infected patients with highly active antiretroviral therapy HIV Med 2000 1 107 115 10.1046/j.1468-1293.2000.00012.x 11737333

[B7] Kempen JH Min YI Freeman WR Holland GN Friedberg DN Dieterich DT Jabs DA Studies of Ocular Complications of ARG Risk of immune recovery uveitis in patients with AIDS and cytomegalovirus retinitis Ophthalmology 2006 113 684 694 10.1016/j.ophtha.2005.10.067 16581429

[B8] Holland GN AIDS and ophthalmology: the first quarter century Am J Ophthalmol 2008 145 397 408 10.1016/j.ajo.2007.12.001 18282490

[B9] National AIDS Control Organisation HIV Sentinel Surveillance and HIV Estimation - A Technical Brief 2008 New Delhi, NACO:Govt. of India

[B10] National AIDS Control Organisation Operational Guidelines for ART Centres 2007 New Delhi, NACO:Govt. of India

[B11] Gharai S Venkatesh P Garg S Sharma SK Vohra R Ophthalmic manifestations of HIV infections in India in the era of HAART: analysis of 100 consecutive patients evaluated at a tertiary eye care center in India Ophthalmic Epidemiol 2008 15 264 271 10.1080/09286580802077716 18780260

[B12] Shah SU Kerkar SP Pazare AR Evaluation of ocular manifestations and blindness in HIV/AIDS patients on HAART in a tertiary care hospital in western India Br J Ophthalmol 2009 93 88 90 10.1136/bjo.2008.149237 18952644

[B13] National AIDS Control Organisation Antiretroviral Therapy Guidelines for HIV-infected Adults and Adolescents including Post-exposure 2007 New Delhi, NACO:Govt. of India

[B14] Jadoon MZ Dineen B Bourne RR Shah SP Khan MA Johnson GJ Gilbert CE Khan MD Prevalence of blindness and visual impairment in Pakistan: the Pakistan National Blindness and Visual Impairment Survey Invest Ophthalmol Vis Sci 2006 47 4749 4755 10.1167/iovs.06-0374 17065483

[B15] World Health Organisation Consultation on development of standards for characterisation of visual loss and visual functioning. WHO/PBL/03.091 2003 Geneva, World Health Organisation

[B16] Jabs DA Ocular manifestations of HIV infection Trans Am Ophthalmol Soc 1995 93 623 683 8719695 PMC1312074

[B17] Lawn SD Myer L Bekker LG Wood R Tuberculosis-associated immune reconstitution disease: incidence, risk factors and impact in an antiretroviral treatment service in South Africa AIDS 2007 21 335 341 10.1097/QAD.0b013e328011efac 17255740

[B18] Chitale AR Cancer and AIDS Indian J Pathol Microbiol 2005 48 151 160 16758653

[B19] Ablashi D Chatlynne L Cooper H Thomas D Yadav M Norhanom AW Chandana AK Churdboonchart V Kulpradist SA Patnaik M Seroprevalence of human herpesvirus-8 (HHV-8) in countries of Southeast Asia compared to the USA, the Caribbean and Africa Br J Cancer 1999 81 893 897 10.1038/sj.bjc.6690782 10555764 PMC2374301

[B20] Murthy G The socioeconomic impact of human immunodeficiency virus/acquired immune deficiency syndrome in India and its relevance to eye care Indian J Ophthalmol 2008 56 395 397 10.4103/0301-4738.42416 18711268 PMC2636145

[B21] Wei LL Park SS Skiest DJ Prevalence of visual symptoms among patients with newly diagnosed cytomegalovirus retinitis Retina 2002 22 278 282 10.1097/00006982-200206000-00004 12055459

[B22] Wohl DA Pedersen S Horst CM van der Routine ophthalmologic screening for cytomegalovirus retinitis in patients with AIDS J Acquir Immune Defic Syndr 2000 23 438 439 10866239 10.1097/00126334-200004150-00013

[B23] Brodrick CD Skiest DJ He Y To Screen or Not to Screen, Can Symptoms Answer the Question: How Efficient is Routine Screening for CMV Retinitis in the HAART Era? Invest Ophthalmol Vis Sci 2003 44 3128

[B24] Heiden D Ford N Wilson D Rodriguez WR Margolis T Janssens B Bedelu M Tun N Goemaere E Saranchuk P Cytomegalovirus retinitis: the neglected disease of the AIDS pandemic PLoS Med 2007 4 e334 10.1371/journal.pmed.0040334 18052600 PMC2100142

